# *Ligilactobacillus salivarius* 2102-15 complete genome sequence data

**DOI:** 10.1016/j.dib.2023.109564

**Published:** 2023-09-12

**Authors:** Andrey V. Karlyshev, Simon Gould

**Affiliations:** Department of Biomolecular Sciences, School of Life Sciences, Chemistry and Farmacy, Faculty of Health, Science, Social Care and Education, Kingston University London, Kingston upon Thames, KT1 2EE, UK

**Keywords:** *Ligilactobacillus salivarius*, Probiotics, Genome sequence, Bacteriocins

## Abstract

The article presents *Ligilactobacillus salivarius* 2102-15 whole genome sequencing data generated by using Illumina and Oxford Nanopore platforms. The genome of the isolate consists of a chromosome and two plasmids. The data on bacteriocin-encoding genes present in the genome were collected through genome annotation and by using a BAGEL4 tool. The advantages and limitations of the approaches are highlighted. The data indicate the presence of different types of bacteriocin and immunity protein-encoding genes on both the chromosome and one of the plasmids. The data obtained represents interest to researchers working in the areas related to whole genome sequencing and analysis, as well as being useful for the identification of novel probiotic bacteria and their biomedical applications.

Specifications Table

**Table:** 

Subject	Biological Sciences; Omics: Genomics
Specific subject area	Genomics, applied microbiology
Type of data	Raw sequencing reads Assembled genome Annotated genome Tables Figures
How the data were acquired	*Ligilactobacillus salivarius* strain 2102-15 was isolated at the National Medical Research Center for Obstetrics, Gynecology and Perinatology named after Academician V.I.Kulakov, Moscow, Russia The sequencing data were generated using Illumina HiSeq 2500 and Oxford Nanopore GridION systems.
Data format	Raw data and analysed data
Description of data collection	Total genomic DNA was extracted using GenElute Bacterial Genomic DNA Kit and sequenced using Illumina NovaSeq 6000and Oxford Nanopore GridION systems generating short and long tools respectively. *De novo* assembly of the reads, including scaffolding and gap-filling was performed using Unicycler v0.4.9b. Whole genome sequence genome annotation was performed by NCBI Prokaryotic Genome Annotation Pipeline (PGAP) v5.3. BAGEL4 tool was used for the generation of data on bacteriocin-encoding genes.
Data source location	Institution: Kingston University London City/Town/Region: Kingston upon Thames County: United Kingdom Latitude and longitude 51.575650 and 0.188520
Data accessibility	Direct URLs to data: Raw data Repository name: National Center for Biotechnology Information (NCBI) Sequence reads were deposited at NCBI SRA database under accession No. PRJNA793583: https://www.ncbi.nlm.nih.gov/sra/PRJNA793583 BioProject https://www.ncbi.nlm.nih.gov/bioproject/PRJNA793583 BioSample https://www.ncbi.nlm.nih.gov/biosample/SAMN24563524 Assemblies Combined records: https://www.ncbi.nlm.nih.gov/nuccore/?term=CP090411:CP090413[accn] 2102-15 chromosome: https://www.ncbi.nlm.nih.gov/nuccore/CP090411.1 plasmid pLS2102-15_1: https://www.ncbi.nlm.nih.gov/nuccore/CP090412.1 plasmid pLS2102-15_2: https://www.ncbi.nlm.nih.gov/nuccore/CP090413.1 Tables and Figures – with the article

## 1 Value of the Data

• The data contain information about the complete genome sequence of *Ligilactobacillus salivarius* 2102-15 and are useful for a potential application of this strain as a probiotic.
• The data would benefit researchers involved in comparative genomics studies.
• The data on bacteriocin-related genes are of particular interest for their exploration for the development of novel antimicrobial strategies for fighting infections caused by multidrug resistant bacteria.
• The data on bacteriocin-encoding genes of both chromosomal and plasmid origin, and a strategy used for their identification, would assist scientists studying the genetic origin and application of these antimicrobial compounds.

## 2 Objective

The aim of this article is to provide data on complete genome sequence of *Ligilactobacillus salivarius* 2102-15 containing a chromosome and two plasmids, as well as raw genome sequencing data including the number and sizes of long and short sequencing reads used for genome sequence assembly, as well as specific details of the genome assembly, including data on bacteriocin-encoding genes.

## 3 Data Description

*Ligilactobacillus salivarius* 2102-15 strain was isolated from vaginal exudate of a healthy woman of reproductive age. *L. salivarius* 2102-15 strain was cultivated in De Man, Rogosa, and Sharpe (MRS) broth and agar (HiMedia, India) under anaerobic conditions. The isolate identification on the genus and species levels as *L. salivarius* was performed by using MALDI-TOF-MS method [1]. The woman was selected during a preventive medical examination among a group of patients of reproductive age planning the birth of a child.

**Fig. 1:**
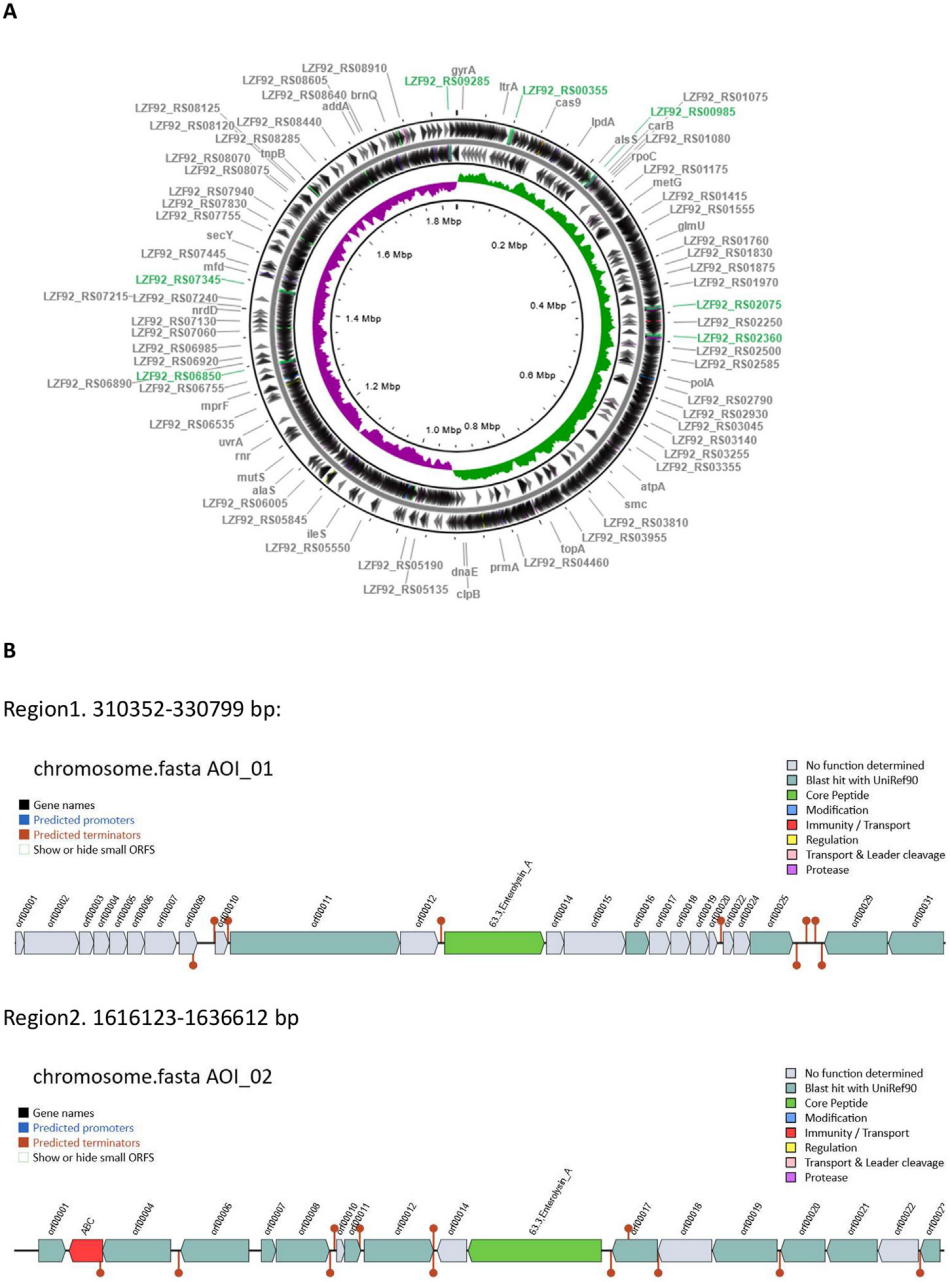
A, circular map *Ligilactobacillus salivarius* 2102-15 chromosome; GC skew+ and GC skew- strains are designated with green and pink respectively. Positions of rRNA are indicated with green color. B, identification of two chromosomal gene clusters containing genes encoding enterolysin-line proteins using BAGEL4 software.

**Fig. 2:**
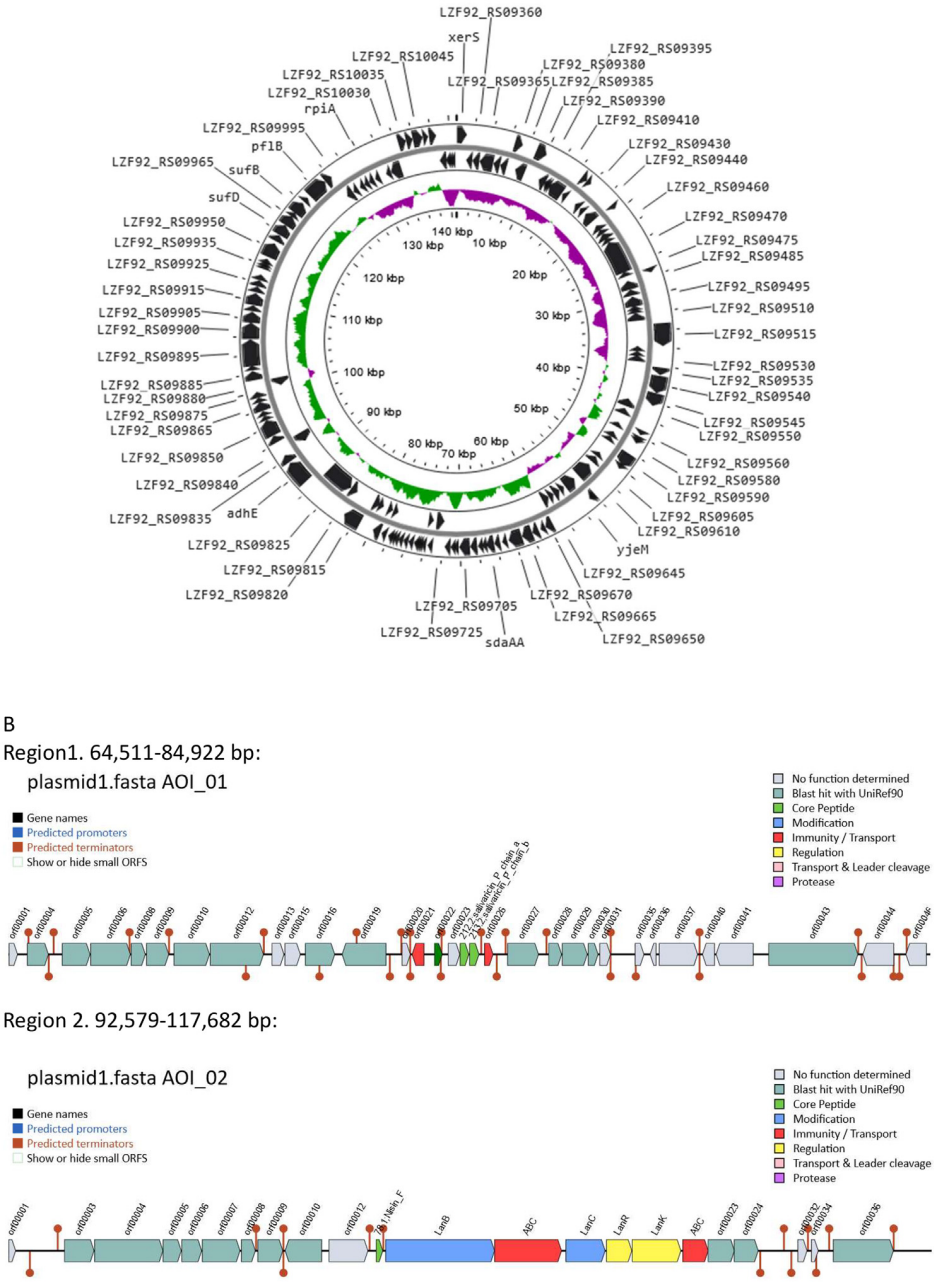
A, genetic map of plasmid plasmid pLS2102-15_1; GC skew+ and GC skew- strains are designated with green and pink respectively. B, identification of two chromosomal gene clusters containing genes encoding enterolysin-line proteins using BAGEL4 software. Two light-green ORFs encode two chain of bacteriocin salvaricin. Dark green ORF 00022 in this cluster encode another bacteriocin, also annotated as such in GenBank. However, unlike the latter, BAGELS failed to identify ORF 00023 as a bacteriocin-encoding gene.

**Fig. 3:**
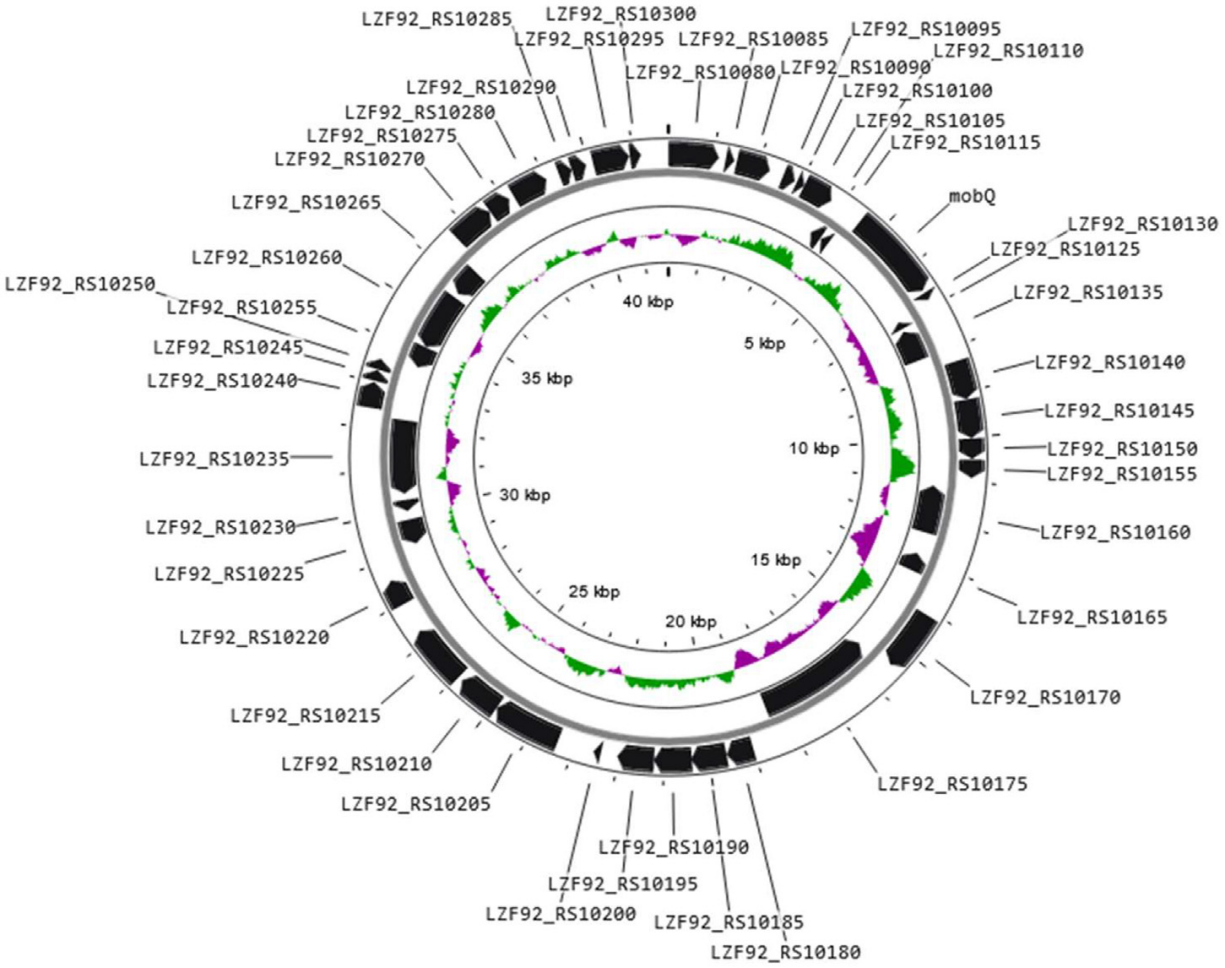
Genetic map of plasmid pLS2102-15_2.

**Fig. 4:**
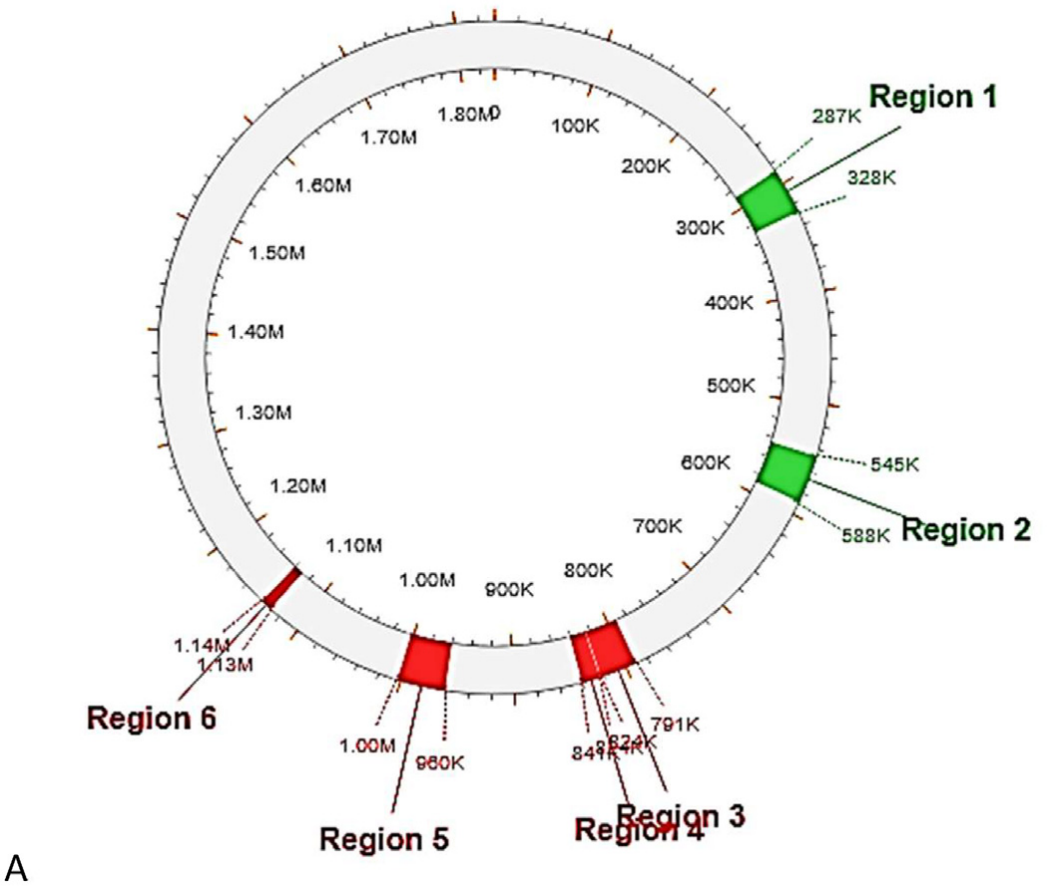
Prophages in the chromosome. Green – intact, red – incomplete.

This article reports data on the complete genome sequence of this isolate containing a chromosome and two plasmids. The data on bacteriocin-related genes found in this genome are provided in more details as they may contribute to specific properties of this isolate. The genome sequence of *Ligilactobacillus salivarius* 2102-15 is available at NCBI GenBank under the accession numbers CP090411:CP090413. The total size of the genome is 2,017,204 bp with an average GC content of 33.07 % (Table 1). In addition to a circular chromosome (1,834,593 bp), the strain contains two circular plasmids (140,826 bp and 41,785 bp) (Figs. 1–3). The data demonstrate the presence of several genes encoding different bacteriocin-related proteins, involved in the production and immunity to these compounds. There are genes encoding compounds similar to salivaricin, nisin and enterolysins. These data (presented in Table 2) were obtained by using two different approaches: *via* keyword searches of the genome annotation generated by NCBI GenBank, and by an application of a bacteriocin gene detection software BAGEL4. Whilst two chromosomal genes encoding enterolysin A were detected by the latter, the corresponding gene products were annotated by GenBank as a ‘phage tail’ protein (locus tag LZF92_01565) and a ‘peptidoglycan DD-metalloendopeptidase family protein’ (locus tag LZF92_08285). The genes encoding these proteins are located at positions 319,667 bp to 321,982 bp and 1,626,121 bp to 1,629,057 bp respectively. The data presented in Fig. 4 demonstrate location of the former in an intact prophage, spanning a region between 287 kb and 328 kb. Additional data regarding genes encoding bacteriocins are presented Table 2 and Figs. 1 and 2 showing chromosome-located genes encoding enterolysin A, and the genes encoding lantibiotics (a and b chains of salvaricin, and nisin) present on a larger plasmid. The lantibiotics and bacteria producing them were found to be useful for fighting infections, especially those caused by multidrug resistance bacteria [2]. There is also an extra bacteriocin-related gene found by PGAP but not by BAGEL4. Overall, the data demonstrate importance of using a combination of different tools for the identification of bacteriocin-related genes.

**Table 1:** Data on complete genome sequence of *Ligilactobacillus salivarius* 2102-15.

Feature	Value
Total genome sequence size, bp GC content, % Total number of CDSs Total number of genes Protein coding genes 5S rRNA gene 16S rRNA genes 28S rRNA genes tRNAs CRISPR arrays Chromosome size, bp Plasmid pLS2102-15_1 size, bp Plasmid pLS2102-15_2 size, bp	2,017,204 33.07 1956 2060 1933 8 7 7 78 1 1,834,593 140,826 41,785

**Table 2:** Selected putative gene products related to bacteriocin biosynthesis and immunity in *Ligilactobacillus salivarius* 2102-15.

Locus tag	Refseq	location	Product size, amino acids	Amino acid sequence start	Product name, by GenBank	Product name, by BAGEL4	Replicon
LZF92_00585	WP_041822591.1	131323..131634	103	MEKVQ	bacteriocin immunity protein	none	chromosome
LZF92_06760	WP_003710547.1	1301004..1301357	117	MERTK	bacteriocin immunity protein	none	
LZF92_01565	WP_011475663.1	319793..321079	729/771^1^	VYDDG	Phage tail protein	Enterolysin A	
LZF92_08285	WP_004564100.1	1626121..1629057 (complement)	978	MYKAG	peptidoglycan DD-metalloendopeptidase family protein	Enterolysin A	
LZF92_09735	WP_003708563.1	73451..73711	87	MNSFS	hypothetical	bacteriocin immunity protein	plasmid pLS2102-15_1
LZF92_09740	WP_041823193.1	73944..74117	57	MNNNF	bacteriocin	ComC; Bacteriocin_IIc	
LZF92_09745	WP_011476663.1	74247..74504	85	MLKKL	bacteriocin	none	
LZF92_09750	WP_003703435.1	74510..74704	64	MMKEF	Blp family class II bacteriocin	salivaricin P, chain a	
LZF92_09755	WP_003703485.1	74722..74928	68	MKNLD	Blp family class II bacteriocin	salivaricin P, chain b	
None	none	75052..75237	64	MMKEF	none	bacteriocin immunity protein	
LZF92_09760	WP_003703471.1	75360..75476	38	MKFEV	bacteriocin	none	
LZF92_09890^2^	WP_014570405.1	102578..102757	59	MSVND	gallidermin/nisin family	nisin_F, lanthipeptide A	

1 Enterolysin sequence detected by BAGEL4 is a part of phage tail protein annotated by GenBank.

2 Nisin biosynthesis and transport involves a number of other gene products not included into this table.

## 4 Experimental Design, Materials and Methods

### 4.1 Sample Preparation, Genome Sequencing and Annotation

The strain was grown on MRS agar (Oxoid, USA) for 24 h at 37 °C in an anaerobic atmosphere (5 %hydrogen, 10 % carbon dioxide and the rest nitrogen). Bacteria were resuspended in Tris-EDTA buffer with lysozyme (0.1 mg/ml) and RNase A (0.1 mg/ml), incubated for 25 min at 37 °C, followed by addition of proteinase K and SDS to 0.1 mg/ml and 0.5 %, respectively. After incubated for at 65 °C for 5 min, genomic DNA was purified using reversible immobilization (SPRI) beads (Beckman, USA) and resuspended in elution buffer (Qiagen, Germany).

The complete genome sequencing was performed by using short and long sequencing reads produced by Illumina and Oxford Nanopore sequencing platforms respectively. Short reads sequencing DNA library was prepared using the Nextera XT library prep kit (Illumina, San Diego, CA) following the manufacturer's protocol. Short reads were generated by using the Illumina NovaSeq 6000 platform. Long reads genomic DNA libraries were prepared with the Oxford Nanopore Technologies (ONT; United Kingdom) SQK-LSK109 kit with the native barcoding EXP-NBD104/114 (ONT) kit. Sequencing was done with a FLO-MIN106 (R.9.4.1) flow cell in a GridION system (ONT). Illumina reads were adapter trimmed using Trimmomatic 0.30 with a sliding window quality cutoff of Q15 [3]. All sequencing reads were assembled by using Unicycler v.0.4.9b [4]. The whole genome assembly coverage was 80.0x. The assembly was annotated by the NCBI Prokaryotic Genome Annotation Pipeline [5] using the best-placed reference protein set and GeneMarkS-21. The complete genome sequence is represented by three circular molecules: chromosome (1,834,593 bp) and two plasmids (140,826 and 41,785 bp), graphic images of which were generated using an on-line Proksee server [6] available at https://proksee.ca/. Gene clusters involved in bacteriocin biosynthesis and immunity were identified using BAGEL4 [7] software available at http://bagel4.molgenrug.nl/. Search for prophages was performed by using an online tool PHASTER [8] available at http://phaster.ca/. The online tools Proksee, BAGEL4 and and PHASTER were run on 04/06/2023, 10/06/2023 and 15/06/2023, respectively. Default parameters were used for all software unless otherwise specified.

## Ethics Statement

Not applicable as none of the experiments described in this report involved any human or animal specimens.

## Data availability

BioProject https://www.ncbi.nlm.nih.gov/bioproject/PRJNA793583.

BioSample https://www.ncbi.nlm.nih.gov/biosample/SAMN24563524.

For other data availability please refer to Specification Table.

## CRediT authorship contribution statement

**Andrey V. Karlyshev:** Data curation, Formal analysis, Methodology, Supervision, Writing – review & editing. **Simon Gould:** Data curation, Writing – review & editing.

## Data Availability

Ligilactobacillus salivarius strain 2102-15 chromosome (Original data) (GenBank).
